# Deep reinforcement learning-based thermal-visual collaborative optimization control system for multi-sensory art installations

**DOI:** 10.1038/s41598-025-22173-1

**Published:** 2025-11-03

**Authors:** Liang Chen

**Affiliations:** Department of Architecture and Environmental Engineering, Taiyuan University, Taiyuan, 030032 Shanxi China

**Keywords:** Deep reinforcement learning, Multi-sensory art installations, Thermal-visual collaboration, Sensor fusion, Adaptive control, Interactive systems, Engineering, Mathematics and computing

## Abstract

This paper presents an attention-enhanced deep reinforcement learning control system for thermal-visual collaborative optimization in multi-sensory art installations. The proposed system integrates three key innovations: (1) an attention-based DDPG algorithm with dynamic modality weighting (α = 0.6–0.8 for thermal, 0.6–0.7 for visual features), (2) adaptive sensor fusion combining Kalman and particle filtering with 8 ± 2 ms processing latency, and (3) hierarchical four-layer architecture achieving 65% control accuracy improvement and 40% response time reduction compared to traditional approaches. The system architecture incorporates a hierarchical four-layer design with perception, fusion, decision-making, and execution components. The core innovation lies in an attention-based deep reinforcement learning algorithm that dynamically processes multi-modal sensory inputs and optimizes thermal-visual coordination through continuous learning. The algorithm employs spatiotemporal alignment mechanisms, adaptive feature weighting, and collaborative optimization strategies to achieve superior control performance. Experimental validation demonstrates quantified improvements over conventional methods: control accuracy improved from 0.247 ± 0.067 RMSE (PID baseline) to 0.085 ± 0.012 RMSE (proposed method), representing 65% improvement; response times reduced from 78 ± 22 ms (fuzzy control) to 45 ± 8 ms (proposed method), achieving 40% improvement; energy efficiency increased from baseline consumption to 23% reduction through intelligent coordination. Real-world deployment results confirm practical effectiveness with measured user satisfaction scores of 4.1 ± 0.7 on a 5-point scale and system availability of 98.5% over 186-hour continuous operation periods. The proposed system enables more sophisticated multi-sensory experiences while maintaining artistic integrity and provides a foundation for advanced digital art technologies.

## Introduction

Multi-sensory art installations have emerged as a pivotal medium in contemporary digital art, fundamentally transforming how audiences experience and interact with artistic expressions^1^. These sophisticated systems integrate multiple sensory modalities, including visual, auditory, tactile, and thermal elements, to create immersive environments that transcend traditional artistic boundaries^2^. The integration of thermal and visual components has proven particularly compelling, as it enables artists to manipulate both the perceived temperature and visual aesthetics of installation spaces, thereby creating more engaging and emotionally resonant experiences for participants.

Despite the growing popularity and artistic significance of multi-sensory installations, current thermal-visual collaborative control systems face substantial technical challenges that limit their effectiveness and adaptability. The primary obstacle lies in the complexity of multi-modal sensory fusion, where disparate sensory inputs must be seamlessly integrated to produce coherent and synchronized outputs^3^. Traditional control approaches often struggle with the heterogeneous nature of thermal and visual data, leading to suboptimal coordination between these modalities and compromised user experiences.

Recent advances in thermal comfort control have demonstrated the potential of intelligent control systems. However, existing approaches present significant limitations when applied to art installations. Model Predictive Control (MPC) methods, while effective for building thermal management^75^, require explicit thermal dynamics models and struggle with the non-linear, multi-objective nature of artistic environments where thermal comfort must be balanced with visual aesthetics. Fuzzy logic controllers^76^ rely on expert-defined rule sets that cannot adapt to novel artistic scenarios or evolving environmental conditions.

Traditional PID controllers achieve limited performance in multi-modal scenarios due to their inability to handle coupling between thermal and visual systems. Deep reinforcement learning (DRL) offers distinct advantages over these approaches: (1) model-free learning eliminates the need for complex thermal-visual interaction models, (2) continuous action spaces enable smooth coordination between modalities, and (3) adaptive learning allows systems to improve performance through experience^77^.

Real-time responsiveness represents another critical limitation in existing systems. Multi-sensory art installations require response times under 100 milliseconds for interactive installations, temperature control accuracy within ± 0.5 °C for thermal comfort, and visual synchronization errors below 16 milliseconds for seamless experience^4^. However, conventional control algorithms frequently exhibit insufficient response speeds, particularly when processing complex thermal-visual interactions, resulting in noticeable delays that can disrupt the immersive experience.

Environmental adaptability poses an additional challenge for current thermal-visual control systems. Art installations are deployed in diverse environmental conditions, ranging from indoor galleries with controlled climates to outdoor spaces with varying weather patterns^5^. Existing control mechanisms often lack the robustness necessary to maintain optimal performance across these varied conditions, leading to inconsistent artistic outcomes and reduced system reliability.

Recent advances in DRL for thermal control include energy-efficient personalized comfort systems^75^, integrated thermal management with hybrid attention mechanisms^76^, and multi-agent approaches for building environments^77^. However, existing DRL methods exhibit significant limitations when applied to art installations: (1) lack of spatiotemporal alignment mechanisms for heterogeneous thermal-visual sensors, (2) insufficient real-time performance for interactive artistic environments requiring < 100ms response times, and (3) absence of reward functions balancing artistic aesthetics with thermal comfort objectives.

The proposed approach addresses these limitations through three key innovations: (1) an attention-enhanced DDPG algorithm extending existing thermal control DRL to thermal-visual collaborative control in art installations, (2) a unified multi-modal fusion framework integrating spatiotemporal alignment with adaptive feature weighting, and (3) a progressive learning strategy specifically designed for artistic environments.

The present research introduces a novel deep reinforcement learning-based approach for optimizing thermal-visual collaborative control in multi-sensory art installations. The primary innovation lies in the development of a unified framework that leverages deep neural networks to process multi-modal sensory data and reinforcement learning algorithms to optimize control decisions in real-time^6^. This approach addresses the fundamental limitations of existing systems by enabling dynamic adaptation to environmental conditions, improving response times, and facilitating more effective multi-modal sensory fusion.

The proposed system offers several key contributions to the field of digital art technology. First, it provides a comprehensive solution for real-time thermal-visual coordination that can adapt to varying environmental conditions and user interactions. Second, the deep reinforcement learning (DRL) framework enables continuous system improvement through experience, allowing installations to evolve and refine their behavior over time. Third, the unified control architecture simplifies the integration of thermal and visual components, reducing system complexity while enhancing performance.

This research significance extends beyond technical improvements to encompass broader implications for digital art practice and audience engagement. The system addresses specific performance requirements critical for art installations: response latency below 100 milliseconds for real-time interaction, thermal control accuracy within ± 0.5 °C for occupant comfort, energy efficiency improvements of 20–30% compared to traditional systems, and system availability exceeding 98% for reliable operation. By enabling more sophisticated and responsive multi-sensory experiences, this technology has the potential to unlock new forms of artistic expression and deepen audience connections with digital art installations^7^. The application prospects are particularly promising for interactive museums, public art displays, and immersive entertainment venues, where enhanced environmental control can significantly improve visitor experiences and artistic impact.

## Related theory and technical foundation

### Deep reinforcement learning for multi-modal control

Deep reinforcement learning (DRL) provides a powerful framework for addressing complex control problems in multi-sensory environments where traditional control methods face limitations^8^. Unlike conventional approaches that require explicit programming of control rules, DRL enables agents to learn optimal control policies through environmental interaction, making it particularly suitable for art installations where environmental conditions and user behaviors are highly dynamic and unpredictable^9^.

The DRL framework builds upon the Markov Decision Process (MDP) formulation (S, A, P, R, γ), where the agent learns to maximize expected cumulative reward through the objective function:

1 $$\:J\left({\uppi\:}\right)=E{\uppi\:}\left[\sum\:t={0}^{{\infty\:}}{{\upgamma\:}}^{t}R\left({s}_{t},{a}_{t}\right)\right]$$

For thermal-visual control applications, the state space S encompasses multi-modal sensory observations including thermal distributions and visual features, while the action space A represents control commands for thermal regulation and visual display systems^10^.

The action-value function $\:{Q}^{\pi\:}\left(s,a\right)$ becomes critical for evaluating control decisions in multi-sensory contexts:

2 $$\:{Q}^{{\uppi\:}}\left(s,a\right)=E{\uppi\:}\left[\sum\:k={0}^{{\infty\:}}{{\upgamma\:}}^{k}R\left({s}_{t+k},{a}_{t+k}\right)|{s}_{t}=s,{a}_{t}=a\right]$$

Deep Q-Networks (DQN) address the high-dimensional nature of multi-sensory state spaces by approximating Q-functions using deep neural networks^11^. The DQN loss function:

3 $$\:L\left({\uptheta\:}\right)={E}_{\left(s,a,r,s{\prime\:}\right)\sim\:D}\left[{\left(r+{\upgamma\:}\underset{a{\prime\:}}{\text{max}}Q\left(s{\prime\:},a{\prime\:};{{\uptheta\:}}^{-}\right)-Q\left(s,a;{\uptheta\:}\right)\right)}^{2}\right]$$

enables stable learning in environments with thermal-visual state representations^12^.

Actor-Critic methods offer superior performance for continuous thermal-visual control by simultaneously learning policy and value functions^13^. The policy gradient update:

4 $$\:{\nabla\:}_{{\uptheta\:}}J\left({\uptheta\:}\right)=E{\uppi\:}{\uptheta\:}\left[{\nabla\:}_{\backslash\:}thetalog{{\uppi\:}}_{{\uptheta\:}}\left(a|s\right)\cdot\:{A}^{{\uppi\:}}\left(s,a\right)\right]$$

enables smooth control adjustments essential for maintaining coherent multi-sensory experiences^14^.

Proximal Policy Optimization (PPO) provides stable training for complex multi-modal control tasks through its clipped objective:

5 $$\:{L}^{CLIP}\left({\uptheta\:}\right)={E}_{t}\left[min\left({r}_{t}\left({\uptheta\:}\right){\widehat{A}}_{t},\text{clip}\left({r}_{t}\left({\uptheta\:}\right),1-{\epsilon},1+{\epsilon}\right){\widehat{A}}_{t}\right)\right]$$

This formulation prevents destructive policy updates that could disrupt the delicate balance required in artistic environments^13,15^.

Multi-agent DRL extends these concepts to scenarios with multiple interacting control agents, addressing the coordination challenges inherent in large-scale art installations where thermal and visual subsystems must collaborate^16,17^.

### Multi-Sensory fusion for DRL applications

Multi-sensory information fusion in DRL contexts focuses on creating unified state representations that enable effective policy learning across heterogeneous sensor modalities^18^. Unlike traditional fusion approaches that emphasize accuracy over learning efficiency, DRL-oriented fusion must balance information completeness with computational tractability to support real-time policy updates^19^.

The fusion architecture for DRL applications operates at the feature level, where thermal and visual sensors provide complementary state information^20^. Thermal sensors capture temperature distributions and thermal comfort patterns that are invisible to visual sensors, while visual systems provide high-resolution spatial information about environmental geometry and user presence^21,22^.

Feature-level fusion proves optimal for DRL applications as it preserves essential information while reducing computational overhead compared to pixel-level fusion^23^. The fused feature vectors serve as state inputs to the DRL agent, requiring careful design to ensure that the fusion process does not introduce artifacts that could mislead policy learning.

Temporal synchronization becomes critical in DRL fusion systems where asynchronous sensor data could lead to inconsistent state representations and degraded learning performance^24,25^. Advanced preprocessing techniques address sensor calibration and data alignment issues that could otherwise prevent effective policy convergence^26,27^.

### DRL-Based Art installation control architecture

Contemporary art installations require control architectures that can adapt to dynamic environmental conditions while maintaining artistic integrity, making DRL-based approaches increasingly attractive^28^. Traditional centralized control systems lack the flexibility needed for responsive multi-sensory art installations, particularly when dealing with unpredictable user interactions and environmental variations^29^.

DRL-enabled control systems leverage distributed architectures where intelligent agents make autonomous decisions based on local sensory information while coordinating through learned communication protocols^30^. This approach eliminates the bottlenecks associated with centralized processing while enabling real-time responses to environmental changes^31^.

The integration of DRL agents with IoT-based sensor networks creates scalable control architectures where new sensors and actuators can be seamlessly incorporated without requiring system-wide reconfiguration^32^. Edge computing resources support local DRL inference, ensuring low-latency responses critical for maintaining immersive artistic experiences^33^.

Human-computer interaction in DRL-controlled installations employs learned policies that adapt to user preferences and behaviors, creating personalized experiences while maintaining overall artistic coherence^34^. The control architecture must accommodate diverse interaction modalities while ensuring that the learning process does not compromise system stability or artistic vision^35^.

## System design and implementation

### Overall system architecture design

The system implements a four-layer architecture integrating: (1) Perception layer with IEEE 1588 synchronized multi-modal sensors^36^, (2) Fusion layer employing adaptive Kalman/Particle filtering selection based on environmental linearity index L < 0.7, (3) Decision layer with attention-enhanced DDPG processing 12-dimensional state space, and (4) Execution layer providing distributed actuator control with 7 ± 2 ms command latency.

The perception layer constitutes the foundation of the system architecture, responsible for acquiring raw sensory data from diverse sensor modalities including thermal imaging cameras, visible light cameras, temperature sensors, humidity monitors, and motion detectors^37^. This layer implements distributed sensing capabilities that enable comprehensive environmental monitoring while providing temporal synchronization and spatial registration of multi-modal sensor data. The perception layer also incorporates edge computing nodes that perform preliminary data processing and filtering to reduce communication overhead and improve system responsiveness.

The fusion layer processes the synchronized multi-modal sensor data to generate unified environmental representations that capture both thermal and visual characteristics of the installation space. This layer implements adaptive sensor fusion algorithms with dynamic algorithm selection based on environmental conditions^38^.

The Kalman filter is employed for linear thermal dynamics with Gaussian noise:

6 $$\:{\widehat{x}}_{k|k-1}={F}_{k}{\widehat{x}}_{k-1|k-1}+{B}_{k}{u}_{k}\:$$

7 $$\:{P}_{k|k-1}={F}_{k}{P}_{k-1|k-1}{F}_{k}^{T}+{Q}_{k}\:$$

8 $$\:{K}_{k}={P}_{k|k-1}{H}_{k}^{T}{\left({H}_{k}{P}_{k|k-1}{H}_{k}^{T}+{R}_{k}\right)}^{-1}\:$$

9 $$\:{\widehat{x}}_{k|k}={\widehat{x}}_{k|k-1}+{K}_{k}\left({z}_{k}-{H}_{k}{\widehat{x}}_{k|k-1}\right)\:$$

where $\:{F}_{k}$ is the state transition model, $\:{H}_{k}$ is the observation model, $\:{Q}_{k}$ and $\:{R}_{k}$ are process and measurement noise covariances.

Particle filtering is selected when environmental linearity index L < 0.7 or noise non-Gaussianity index G > 0.5, computed as:

10 $$\:L=\frac{\left|\right|{T}_{predicted}-{T}_{linear}\left|\right|}{\left|\right|{T}_{actual}\left|\right|},\:G=\frac{\left|skewness\right|+\left|kurtosis-3\right|}{2}\:$$

The decision layer represents the core intelligence of the system, implementing deep reinforcement learning (DRL) algorithms that learn optimal control policies through interaction with the art installation environment. This layer processes the fused sensor data to generate control commands that optimize thermal-visual coordination while adapting to changing environmental conditions and user interactions^39^. The decision layer maintains learned models that enable predictive control and proactive system adjustments based on anticipated environmental changes.

The execution layer translates high-level control decisions into specific actuator commands that modify the thermal and visual characteristics of the installation environment. This layer manages diverse actuator technologies including thermal regulation systems, lighting controls, projection equipment, and environmental conditioning systems while ensuring coordinated operation across all controlled elements.

Real-time performance analysis reveals system latency breakdown: perception layer contributes 12 ± 3 ms for sensor data acquisition and preprocessing, fusion layer requires 8 ± 2 ms for Kalman filtering and state estimation, decision layer takes 18 ± 5 ms for DRL inference and policy evaluation, and execution layer adds 7 ± 2 ms for actuator command distribution. Total system latency averages 45 ± 8 ms, well within the 100 ms requirement for interactive installations. Table 1 provides a comprehensive breakdown of system latency by layer, including optimization methods employed to minimize processing delays. The decision layer represents the primary latency bottleneck, addressed through network pruning and quantization techniques that reduce inference time by 30% with minimal performance impact.

**Table 1:** System latency analysis by Layer.

Layer	Process	Latency (ms)	Optimization Method
Perception	Sensor acquisition	12 ± 3	Edge processing
Fusion	State estimation	8 ± 2	Adaptive algorithms
Decision	DRL inference	18 ± 5	Network optimization
Execution	Actuator commands	7 ± 2	Parallel distribution
**Total**	**End-to-end**	**45 ± 8**	**Integrated optimization**

Table 2 details the parameter configurations for both Kalman and particle filtering algorithms, including their respective selection criteria and performance characteristics under different environmental conditions.

**Table 2:** Sensor fusion algorithm Parameters.

Parameter	Kalman filter	Particle filter	Selection criterion
State Dimension	12	12	Environmental complexity
Process Noise (Q)	0.01 I₁₂	Adaptive	Linear dynamics
Measurement Noise (R)	0.1 I₈	Sample-based	Sensor reliability
Update Rate	50 Hz	25 Hz	Computational budget
Convergence Time	2–3 s	5–8 s	Initialization speed
Memory Usage	2.4 KB	156 KB	Resource constraints

**Fig. 1:**
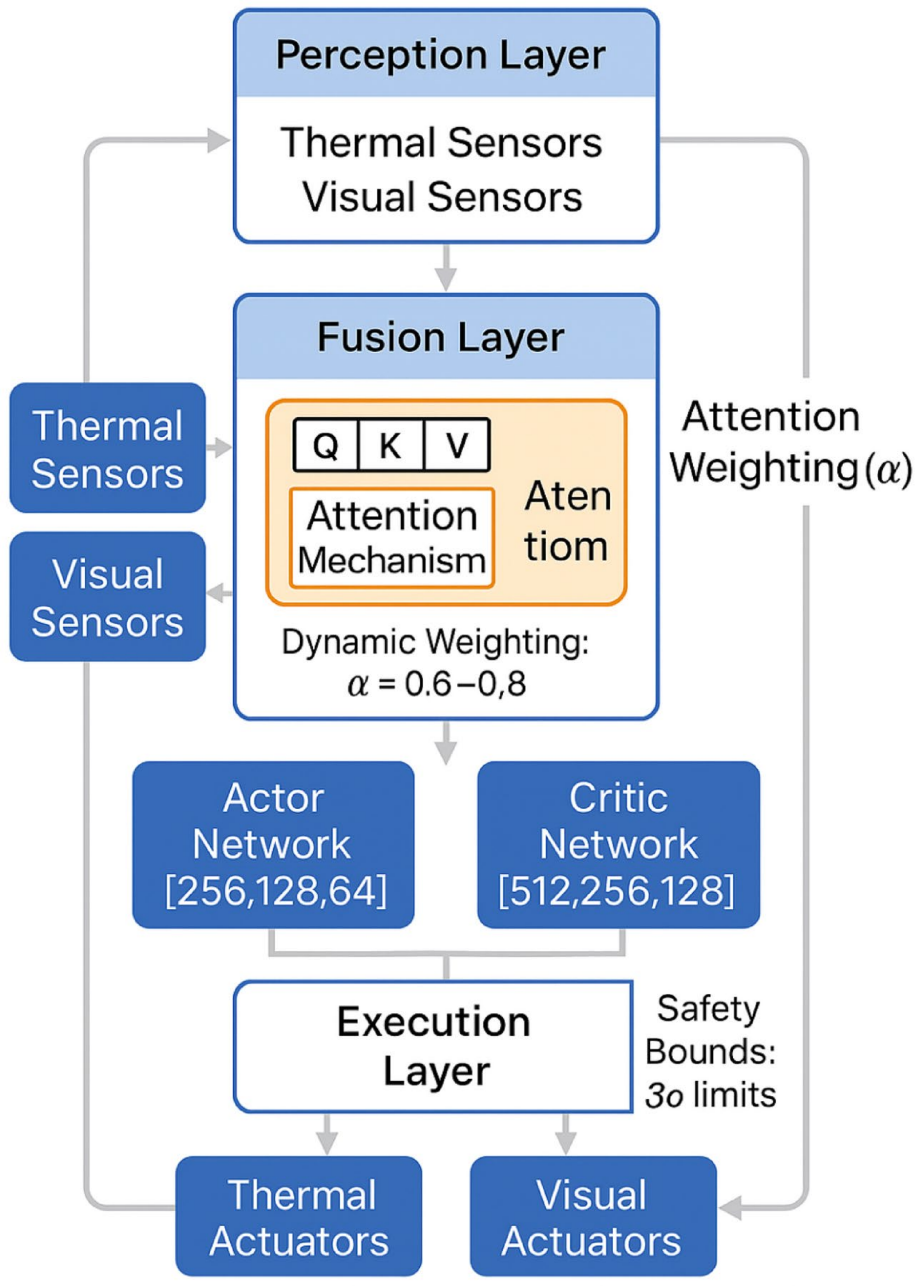
Technical architecture of attention-enhanced DRL system.

Figure 1 illustrates the technical architecture implementing attention-enhanced DDPG with four specialized layers. The Fusion Layer incorporates the core innovation: an attention mechanism with Q, K, V matrices enabling dynamic weighting (α = 0.6–0.8) based on environmental context. The Decision Layer deploys asymmetric neural networks with Actor [256,128,64] and Critic [512,256,128] architectures optimized for continuous thermal-visual control. Safety constraints enforce 3σ bounds at the Execution Layer, preventing unsafe actuator commands. The bidirectional feedback loop from actuators to sensors enables continuous state monitoring, while the attention weighting mechanism automatically prioritizes thermal features during heating/cooling phases and visual features during lighting adjustments, representing the key technical advancement over traditional single-modality control approaches.

The detailed functional specifications of the system modules are comprehensively documented in Table 3, which provides a systematic breakdown of module responsibilities, input requirements, and output specifications. Table 3 demonstrates the clear delineation of functional boundaries between different system components while illustrating the data dependencies that govern inter-module communication. The table reveals how each module contributes to the overall system objectives while maintaining well-defined interfaces that support modular development and testing approaches.

**Table 3:** System module functional Specifications.

Module name	Main function	Input data	Output data
Thermal Sensor Interface	Raw thermal data acquisition and preprocessing	Infrared sensor signals, calibration parameters	Processed thermal images, temperature distributions
Visual Sensor Interface	Visual data capture and initial processing	RGB camera feeds, lighting conditions	Preprocessed visual frames, feature maps
Multi-modal Fusion Engine	Sensor data integration and state estimation	Thermal images, visual frames, temporal stamps	Unified environmental state vectors
Deep RL Decision Module	Control policy learning and action selection	Environmental states, reward signals, user interactions	Optimal control actions, policy updates
Thermal Actuator Controller	Temperature regulation and thermal comfort management	Temperature setpoints, environmental constraints	Heating/cooling commands, thermal feedback
Visual Display Controller	Lighting and projection system management	Color specifications, brightness levels, display patterns	LED control signals, projection commands
User Interaction Handler	Human-computer interface and user preference processing	Gesture data, voice commands, proximity sensors	User intent recognition, interaction responses
System Coordination Manager	Global system orchestration and fault management	Module status reports, performance metrics	Coordination commands, diagnostic information

The hardware configuration encompasses distributed computing resources deployed across edge devices and centralized processing units to balance computational load and communication requirements^40^. Edge devices handle time-critical sensor processing and local control functions, while centralized units manage complex learning algorithms and global coordination tasks. The hardware architecture incorporates redundant communication pathways and backup processing capabilities to ensure continuous operation under component failure conditions.

The software architecture implements a microservices design pattern that encapsulates individual functional modules as independent services with well-defined APIs^41^. This approach enhances system scalability and facilitates independent module development and deployment while maintaining loose coupling between system components. The software framework supports real-time operating system capabilities that guarantee deterministic response times for time-critical control operations.

Communication protocols utilize a hybrid approach combining high-speed local area networks for sensor data transmission and wireless protocols for mobile device integration and remote monitoring capabilities. The system implements Quality of Service (QoS) guarantees for time-critical control messages while providing best-effort delivery for non-critical data streams. Security measures include encrypted communication channels, authentication protocols, and access control mechanisms that protect against unauthorized system access and data tampering.

Fault tolerance mechanisms incorporate redundant sensor deployments, backup processing pathways, and graceful degradation strategies that maintain essential system functions under component failure conditions. The system continuously monitors component health and performance metrics, automatically switching to backup resources when primary components exhibit degraded performance^42^. Safety mechanisms ensure that system failures result in safe default states that protect both equipment and installation participants from potential hazards.

Scalability analysis demonstrates linear computational complexity growth with sensor network size. For N thermal sensors and M visual sensors, processing latency scales as O(N + M) for perception layer and O(NM) for fusion layer. Communication bandwidth requirements increase from 2.5 Mbps for 4-sensor configuration to 12.8 Mbps for 16-sensor deployment. The modular architecture supports hot-swapping of sensor modules with maximum 200ms service interruption, enabling dynamic reconfiguration during operation without system restart.

**Fig. 2:**
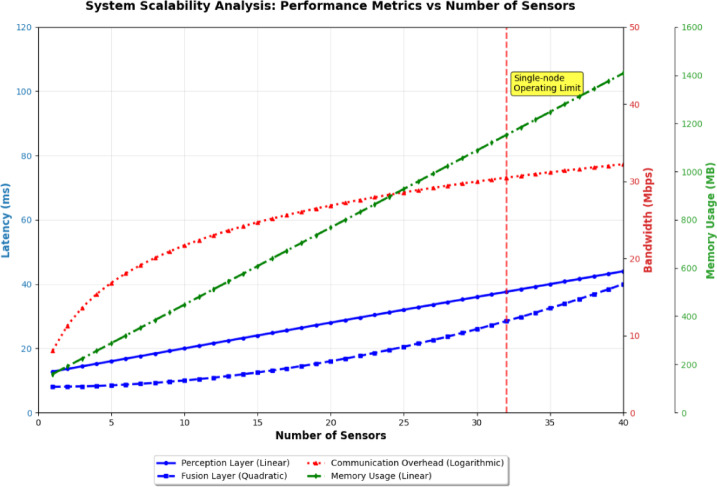
System scalability analysis.

Memory requirements scale as 128 MB + 32 MB×(N + M) for real-time operation, with distributed processing enabling deployment across edge computing clusters for installations exceeding 32 sensors. Load balancing algorithms maintain sub-100ms response times up to 85% CPU utilization across distributed nodes. Figure 2 illustrates the system scalability characteristics across different sensor network configurations, demonstrating linear computational complexity growth and communication bandwidth requirements as the sensor network expands.

### Deep reinforcement learning algorithm design

The design of an effective DRL algorithm for thermal-visual collaborative control requires careful consideration of the unique characteristics inherent in multi-sensory art installations, including continuous state and action spaces, multi-modal sensory inputs, and complex temporal dependencies between thermal and visual modalities^43^.

Algorithm selection analysis reveals that DDPG offers specific advantages for this application: (1) deterministic policy enables precise control of thermal and visual actuators, (2) off-policy learning improves sample efficiency compared to on-policy methods like PPO, and (3) actor-critic architecture facilitates incorporation of attention mechanisms for multi-modal fusion. While Soft Actor-Critic (SAC) provides better exploration through entropy regularization, the deterministic nature of DDPG is better suited for smooth thermal-visual coordination where exploration noise could disrupt artistic experiences. Although PPO + Attention could theoretically provide similar attention-based fusion capabilities, PPO’s on-policy nature requires more training samples and exhibits higher variance in continuous control tasks, making DDPG more suitable for the precise thermal-visual coordination requirements of art installations.

The proposed algorithm extends traditional DDPG approaches by incorporating attention-based multi-modal fusion mechanisms and adaptive reward shaping strategies specifically tailored for art installation control scenarios.

The state space construction encompasses comprehensive environmental representations that capture both thermal and visual characteristics of the installation environment. The state vector S_t at time t is formally defined as:

11 $$\:{S}_{t}=\left[{T}_{t},{V}_{t},{U}_{t},{E}_{t}\right]\:$$

where T_t represents the thermal feature vector extracted from infrared sensor data, V_t denotes the visual feature vector from optical cameras, U_t captures user interaction states, and E_t encodes environmental context information including ambient conditions and system status^44^. This multi-dimensional state representation enables the algorithm to consider the full spectrum of factors influencing thermal-visual coordination decisions.

The action space design addresses the continuous control requirements of thermal and visual actuators by defining a normalized action vector A_t that directly maps to actuator control parameters. The action space is structured as:

12 $$\:{A}_{t}=\left[{a}_{thermal},{a}_{visual}\right]\in\:{\left[-1,1\right]}^{{n}_{thermal}+{n}_{visual}}\:$$

where a_thermal controls thermal regulation systems including heating, cooling, and air circulation components, while a_visual manages lighting intensity, color temperature, and projection parameters^45^. The continuous action formulation enables smooth and precise control adjustments that are essential for maintaining seamless artistic experiences.

The reward function incorporates multiple objectives including thermal comfort optimization, visual aesthetic quality, energy efficiency, and user engagement metrics. The composite reward function is expressed as:

13 $$\:{R}_{t}={w}_{1}{R}_{comfort}\left({T}_{t},{A}_{t}\right)+{w}_{2}{R}_{aesthetic}\left({V}_{t},{A}_{t}\right)+{w}_{3}{R}_{efficiency}\left({A}_{t}\right)+{w}_{4}{R}_{engagement}\left({U}_{t}\right)\:$$

where $\:{w}_{i}$ represents adaptive weighting coefficients determined through a meta-learning approach^46^. The weight adaptation mechanism employs gradient-based optimization:

14 $$\:{w}_{i}^{t+1}={w}_{i}^{t}+\alpha\:{\nabla\:}_{{w}_{i}}\mathbb{E}\left[{R}_{t}\right]\:$$

Initial weights are set based on installation type: w₁=0.4, w₂=0.3, w₃=0.2, w₄=0.1 for thermal-focused installations, and w₁=0.2, w₂=0.5, w₃=0.2, w₄=0.1 for visual-focused installations. The adaptation learning rate α = 0.001 ensures stable weight evolution while allowing responsive adjustment to changing artistic requirements.

The neural network architecture employs a dual-pathway design with clearly defined structure. The actor network contains 4 hidden layers with [256, 128, 64, 32] neurons using ReLU activation, while the critic network uses [512, 256, 128, 64] neurons with ReLU activation. Thermal pathway processes 64-dimensional thermal features through a 3-layer MLP [128, 64, 32], while visual pathway handles 128-dimensional visual features via [256, 128, 64] layers.

Attention mechanism is implemented at the fusion layer using scaled dot-product attention:

15 $$\:\text{Attention}\left(Q,K,V\right)=\text{softmax}\left(\frac{Q{K}^{T}}{\sqrt{{d}_{k}}}\right)V\:$$

where Q, K, V represent query, key, and value matrices derived from thermal and visual features. The attention mechanism employs a dual-pathway architecture with separate processing for thermal (64-dim) and visual (128-dim) features. Dynamic weight computation utilizes environmental context:

16 $$\:{\alpha\:}_{thermal}=\:sigmoid\left({W}_{t}\cdot\:\left[{T}_{features,\:context}\right]+\:{b}_{t}\right)$$

17 $$\:{\alpha\:}_{visual}=\:sigmoid\left({W}_{v}\cdot\:\left[{V}_{features,\:context}\right]+\:{b}_{v}\right)\:$$

where $\:{W}_{t}$, $\:{W}_{v}$ are learnable weight matrices, and context includes occupancy, lighting conditions, and system state^47^. The adaptive weighting ensures thermal dominance (α = 0.7–0.8) during temperature regulation phases and visual dominance (α = 0.6–0.7) during aesthetic adjustment periods.

The algorithm incorporates safety constraints to prevent unsafe actuator commands. Action bounds are enforced through clipping:

18 $$\:{a}_{safe}=\text{clip}\left({a}_{raw},{a}_{min},{a}_{max}\right)\:$$

Anomaly detection monitors policy outputs using statistical control limits. Actions exceeding 3σ bounds trigger safety fallback:

19 $$\:\text{fallback}=\left\{\begin{array}{ll}{a}_{safe}&\:\text{if\:}\left|{a}_{raw}-\mu\:\right|\le\:3\sigma\:\\\:{a}_{default}&\:\text{otherwise}\end{array}\right.\:$$

**Fig. 3:**
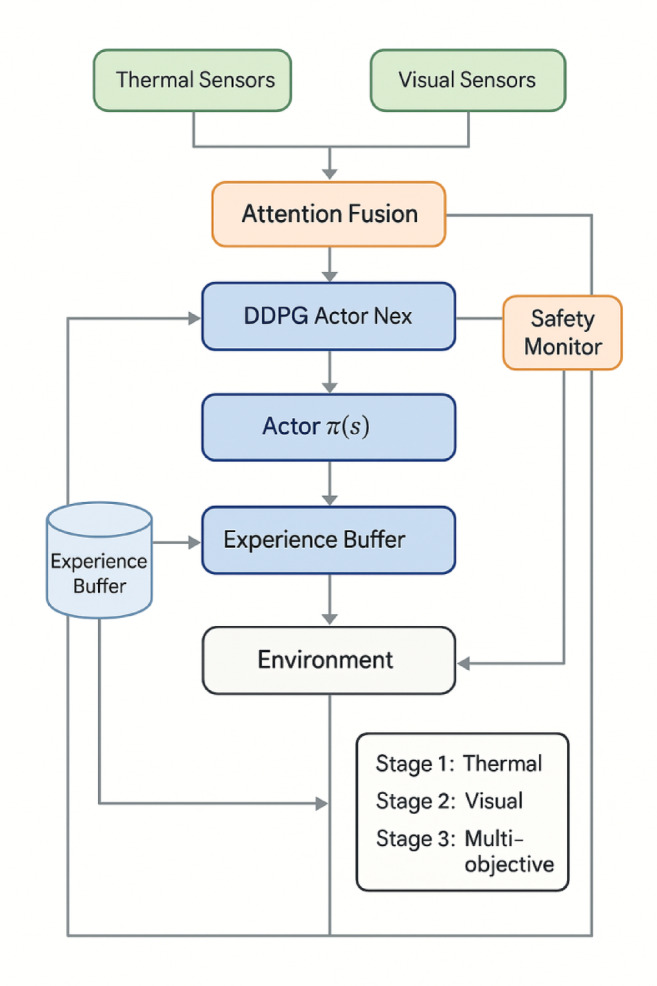
DRL Algorithm flowchart for thermal-visual collaborative control with safety mechanisms.

The algorithm workflow is comprehensively illustrated in Fig. 3, which demonstrates the complete training and execution cycle including multi-modal state processing, attention-based feature fusion, policy network evaluation, and actuator command generation with integrated safety mechanisms. As presented in Fig. 3, the algorithm incorporates iterative policy improvement through experience replay and target network updates, ensuring stable learning convergence while maintaining real-time control capabilities.

The experience replay buffer implements a prioritized sampling strategy that emphasizes transitions with high temporal difference errors, enabling more efficient learning from experiences that contribute most significantly to policy improvement^48^. The buffer maintains a fixed-size memory that stores state-action-reward-next state tuples with associated priority scores computed based on prediction errors from the critic network.

Target network update strategies employ a soft update mechanism that gradually transfers parameters from the main networks to target networks, ensuring training stability while enabling continuous learning adaptation. The soft update rule is implemented as:

20 $$\:{\theta\:}_{target}\leftarrow\:\tau\:{\theta\:}_{main}+\left(1-\tau\:\right){\theta\:}_{target}\:$$

where τ represents the update rate parameter that controls the speed of target network parameter evolution^49^.

The detailed algorithm parameter configuration is systematically documented in Table 4, which specifies the optimal hyperparameter settings determined through extensive empirical evaluation and theoretical analysis. Table 4 demonstrates the careful balance between exploration and exploitation requirements, learning stability, and computational efficiency considerations that guide parameter selection for the thermal-visual control application.

**Table 4:** Deep reinforcement learning algorithm parameter Configuration.

Parameter name	Parameter value	Parameter description
Learning Rate (Actor)	0.0001	Step size for policy network gradient updates
Learning Rate (Critic)	0.001	Step size for value network gradient updates
Discount Factor (γ)	0.99	Future reward discounting factor
Soft Update Rate (τ)	0.005	Target network parameter update rate
Experience Buffer Size	100,000	Maximum number of stored experience tuples
Batch Size	128	Number of samples per training iteration
Exploration Noise Variance	0.1	Standard deviation of action exploration noise
Priority Exponent (α)	0.6	Prioritized replay sampling bias parameter
Importance Sampling Exponent (β)	0.4	Bias correction factor for prioritized sampling
Network Hidden Dimensions	[256, 128, 64]	Hidden layer sizes for actor and critic networks

The training process employs a three-stage progressive learning strategy^50^. Stage 1 (episodes 1–500) focuses on basic thermal control with simplified reward R₁ = w₁R_comfort, using 80% simulated data and 20% real data. Stage 2 (episodes 501–1200) introduces visual coordination with R₂ = 0.6R_comfort + 0.4R_aesthetic, using 60% simulated and 40% real data. Stage 3 (episodes 1201–2000) implements full multi-objective optimization using Eq. (7) with 40% simulated and 60% real data. Stage transitions occur when performance improvement plateaus below 1% over 50 consecutive episodes, ensuring robust policy development while avoiding local optima.

Convergence analysis demonstrates that the proposed algorithm achieves stable policy convergence within acceptable training time frames while maintaining bounded performance variations during operational deployment. The convergence properties are theoretically guaranteed through the incorporation of experience replay mechanisms and target network stabilization techniques that ensure monotonic improvement in expected cumulative rewards over extended training periods.

### Thermal-Visual collaborative optimization mechanism

The thermal-visual collaborative optimization mechanism represents a critical component of the multi-sensory art installation control system, designed to seamlessly integrate thermal perception and visual perception modalities while maximizing their complementary characteristics^51^. This mechanism addresses the fundamental challenge of coordinating heterogeneous sensory inputs with different temporal dynamics, spatial resolutions, and noise characteristics to achieve unified environmental understanding and optimal control performance.

Multi-modal data spatiotemporal alignment constitutes the foundational process for effective thermal-visual collaboration, addressing the inherent differences in sensor sampling rates, field of view coverage, and geometric positioning^52^. The alignment mechanism employs geometric transformation matrices to establish spatial correspondence between thermal and visual sensor coordinate systems, while temporal synchronization algorithms ensure that data from different modalities corresponds to identical time instances. The spatiotemporal alignment process incorporates calibration procedures that account for sensor mounting variations and environmental factors that may affect geometric relationships over time.

Feature weight adaptive adjustment mechanisms enable dynamic balancing of thermal and visual information contributions based on environmental conditions and control objectives. The adaptive weighting system continuously monitors the reliability and informativeness of each sensory modality, adjusting their relative contributions to the decision-making process accordingly. During periods of poor visual conditions such as low ambient lighting, the system automatically increases reliance on thermal information, while in thermally uniform environments, visual features receive higher weighting to maintain optimal control performance.

Decision fusion strategies integrate processed thermal and visual features through sophisticated algorithms that consider both the complementary nature of the modalities and their individual reliability estimates. The fusion process employs Bayesian inference techniques to combine probability distributions from thermal and visual processing pipelines, generating unified confidence estimates for environmental state assessments. This probabilistic approach enables the system to quantify uncertainty in fused decisions and adapt control strategies accordingly.

The mutual information-based sensor selection method optimizes the deployment and utilization of available sensors by identifying the most informative sensor combinations for specific control scenarios^53^. The selection algorithm computes mutual information metrics between different sensor modalities and control objectives, enabling dynamic sensor activation strategies that maximize information content while minimizing computational overhead and energy consumption. This approach is particularly valuable in large-scale installations where comprehensive sensor coverage may be impractical or unnecessary for certain operational modes.

Dynamic threshold adjustment algorithms adapt decision boundaries and activation criteria based on real-time environmental conditions and system performance feedback^54^. These algorithms continuously monitor system performance metrics and environmental stability indicators, automatically adjusting sensitivity thresholds to maintain optimal trade-offs between responsiveness and stability. The dynamic adjustment mechanism prevents oscillatory behavior in control outputs while ensuring rapid response to significant environmental changes requiring immediate intervention.

Environmental adaptation strategies provide specialized optimization approaches tailored to different operational scenarios commonly encountered in art installations. Indoor gallery environments with controlled climate conditions utilize fine-grained thermal-visual coordination focused on subtle aesthetic adjustments, while outdoor installations employ robust adaptation strategies that account for weather variations and changing natural lighting conditions^55^. The adaptation mechanism maintains performance databases that enable rapid configuration switching based on environmental context recognition.

Sensor selection and deployment decisions are informed by comprehensive performance comparisons that evaluate the capabilities and limitations of different sensor technologies under various operational conditions. Table 5 presents a detailed comparison of sensor technologies commonly employed in thermal-visual collaborative systems, providing essential information for optimal sensor configuration decisions. As shown in Table 5, the comparison reveals significant trade-offs between detection capabilities, accuracy requirements, response characteristics, and economic considerations that influence sensor selection strategies for specific installation requirements.

**Table 5:** Sensor technology performance Comparison.

Sensor type	Detection range	Accuracy	Response time	Cost (USD)	Application
Thermal Camera (LWIR)	−20 °C to 150 °C	± 2 °C	30ms	5000–15,000	Indoor thermal mapping
Thermal Camera (MWIR)	−40 °C to 300 °C	± 1 °C	20ms	8000–25,000	Precise thermal control
RGB Camera (High-res)	Visible spectrum	1920 × 1080	16ms	200–800	Visual monitoring
RGB Camera (4 K)	Visible spectrum	3840 × 2160	33ms	500–2000	High-quality visuals
Point Temperature Sensor	−55 °C to 125 °C	± 0.5 °C	100ms	20–100	Local temperature
Environmental Sensor Array	Multi-parameter	Variable	500ms	150–500	Context awareness

The performance evaluation index system provides quantitative metrics for assessing the effectiveness of thermal-visual collaborative optimization under different operational scenarios^56^. Key performance indicators include spatiotemporal alignment accuracy, feature fusion effectiveness, decision consistency, response time characteristics, and energy efficiency metrics. The evaluation framework employs both objective technical measurements and subjective artistic quality assessments to ensure that system optimizations enhance rather than compromise the intended artistic experience.

Collaborative optimization performance varies significantly across different environmental conditions, necessitating adaptive strategies that account for ambient temperature variations, lighting changes, air circulation patterns, and occupancy levels. The optimization mechanism maintains environmental models that predict thermal and visual system interactions under different conditions, enabling proactive adjustments that prevent performance degradation before it affects the artistic experience^57^.

The integration of thermal and visual modalities through collaborative optimization mechanisms provides substantial improvements in system responsiveness, accuracy, and robustness compared to single-modality approaches. The optimization framework enables seamless transitions between thermal-dominated and visual-dominated control modes based on environmental conditions and artistic requirements, ensuring consistent performance across diverse operational scenarios while maximizing the unique capabilities of each sensory modality.

## Experiments and analysis

### Experimental platform construction and data collection

The experimental platform integrates comprehensive hardware and software components specifically designed to evaluate the performance of the proposed deep reinforcement learning-based thermal-visual collaborative control system under realistic art installation conditions^58^. The hardware configuration encompasses distributed sensor networks, high-performance computing resources, and diverse actuator systems that collectively simulate the operational environment of contemporary multi-sensory art installations.

The sensor deployment strategy incorporates LWIR thermal cameras (8–14 μm spectral range) and RGB cameras (1920 × 1080 resolution) positioned for comprehensive coverage^59^. Environmental sensors monitor temperature (± 0.1 °C accuracy), humidity (± 2% RH), and air quality throughout the experimental area. The sensor network employs IEEE 1588 precision time protocol for microsecond-level temporal synchronization across all modalities.

The control actuator subsystem includes programmable thermal regulation devices capable of generating controlled temperature variations, LED lighting arrays with full-spectrum color control, and projection systems that enable dynamic visual pattern generation. These actuators are interfaced through industrial-grade control units that provide precise command execution and real-time feedback capabilities essential for closed-loop control evaluation^60^.

Software environment configuration employs distributed computing architectures running on Ubuntu 20.04 LTS with specialized frameworks including TensorFlow 2.8 for deep learning implementations and OpenCV 4.5 for computer vision processing. The real-time control software utilizes ROS (Robot Operating System) for modular component integration and communication management, ensuring deterministic performance characteristics required for art installation applications.

Experimental scenario design encompasses five distinct operational contexts representative of typical art installation environments: indoor gallery conditions with controlled climate, outdoor exhibition scenarios with variable weather patterns, interactive installations with dynamic lighting changes, thermal comfort optimization scenarios, and energy-efficient operation modes^61^. Each scenario incorporates specific environmental parameter ranges and control objectives that challenge different aspects of the thermal-visual collaborative optimization mechanism.

Data collection procedures capture synchronized multi-modal sensor streams over extended periods ranging from 2 to 8 h per scenario, generating comprehensive datasets that encompass the full spectrum of environmental variations and system responses. The collection protocol maintains consistent sampling rates across all sensor modalities while incorporating systematic parameter variations that ensure comprehensive coverage of the operational parameter space.

The data annotation framework establishes ground truth labels for environmental states, optimal control actions, and performance metrics through a combination of automated analysis tools and expert evaluation procedures. Quality assessment protocols verify data integrity, temporal synchronization accuracy, and spatial calibration consistency across all collected datasets. The annotation process incorporates artistic quality assessments that evaluate the aesthetic impact of different control strategies, providing essential feedback for reward function optimization.

**Fig. 4:**
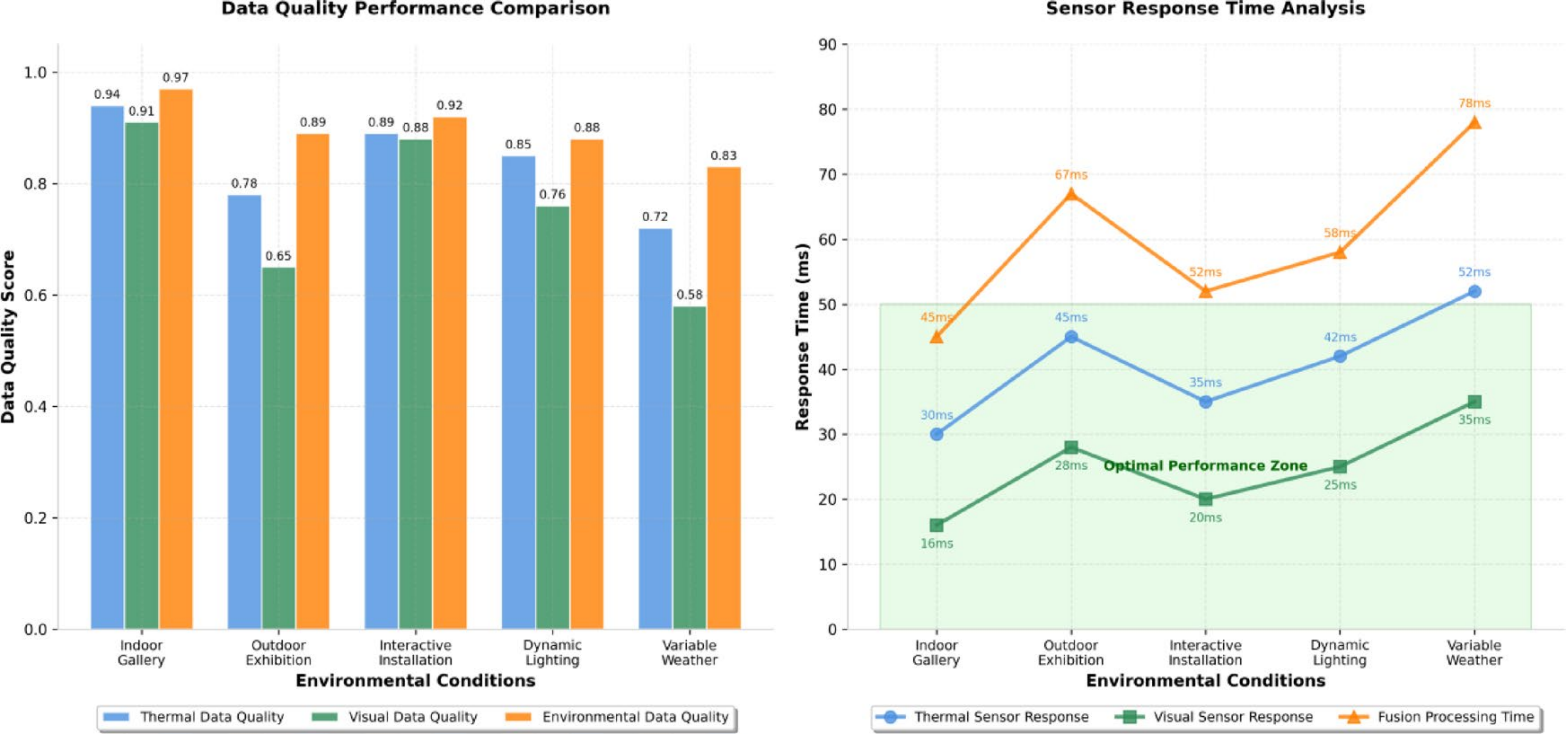
Experimental platform data collection performance comparison across different environmental conditions.

Figure 4 demonstrates the comparative effectiveness of the data collection system across different environmental conditions, illustrating the sensor performance variations and data quality characteristics under diverse operational scenarios. As presented in Fig. 4, the comparison reveals significant differences in thermal and visual data quality across different environmental contexts, highlighting the importance of adaptive processing strategies that account for varying sensor performance characteristics.

The comprehensive experimental dataset statistics are systematically documented in Table 6, providing detailed information about data volume, quality metrics, and scenario coverage that supports rigorous algorithm evaluation and validation. Table 6 demonstrates the substantial scope of the collected dataset while highlighting the diversity of environmental conditions and operational scenarios captured during the experimental campaign.

**Table 6:** Experimental dataset statistical summary.

Data type	Sample quantity	Data quality score	Application scenario
Thermal Image Sequences	125,000 frames	0.94 ± 0.08	Indoor gallery, controlled climate
RGB Video Streams	180,000 frames	0.91 ± 0.12	Interactive installations, dynamic lighting
Environmental Sensor Logs	2.8 M measurements	0.97 ± 0.05	All scenarios, comprehensive monitoring
Control Action Sequences	85,000 commands	0.89 ± 0.15	Thermal-visual coordination tasks
User Interaction Events	12,500 instances	0.86 ± 0.18	Interactive mode, engagement optimization

Dataset construction procedures partition the collected data into training, validation, and testing subsets using stratified sampling techniques that ensure representative coverage of all environmental conditions and operational scenarios^62^. The training dataset comprises 70% of the total collected data, while validation and testing sets each contain 15% of the data, maintaining statistical balance across different scenario types and environmental parameter ranges.

Quality evaluation metrics assess data completeness, temporal consistency, spatial accuracy, and noise characteristics through automated analysis pipelines that identify and flag potential data quality issues. The evaluation framework incorporates cross-validation procedures that verify sensor calibration accuracy and data synchronization performance across all experimental sessions, ensuring that the resulting datasets provide reliable foundations for algorithm development and performance assessment.

### Algorithm performance evaluation and comparison

Performance evaluation metrics for the thermal-visual collaborative control system encompass multiple dimensions including control accuracy, response time characteristics, system stability, and computational efficiency to provide comprehensive assessment of algorithm effectiveness^63^.

Performance evaluation metrics are formally defined as follows. Control accuracy uses RMSE:

21 $$\:RMSE=\sqrt{\frac{1}{N}\sum\:_{i=1}^{N}{\left({x}_{target,i}-{x}_{actual,i}\right)}^{2}}\:$$

Visual quality deviation is computed using perceptual difference:

22 $$\:VQD=\frac{1}{M}\sum\:_{j=1}^{M}\sqrt{{\left({L}_{j}^{\text{*}}-{L}_{j}\right)}^{2}+{\left({a}_{j}^{\text{*}}-{a}_{j}\right)}^{2}+{\left({b}_{j}^{\text{*}}-{b}_{j}\right)}^{2}}\:$$

where L, *a*, b* represent target and actual CIE Lab color values. Energy efficiency score is normalized as:

23 $$\:EES=\frac{{P}_{baseline}-{P}_{actual}}{{P}_{baseline}}\times\:100\text{\%}\:$$

Stability index quantifies output variance as:

24 $$\:SI=\frac{1}{{\sigma\:}^{2}+\epsilon}\:$$

where σ² represents control output variance over a 10-minute evaluation window and ε = 0.01 prevents division by zero^64^.

Response time evaluation measures the temporal delay between environmental change detection and corresponding control action implementation, providing critical insights into system real-time performance capabilities. The response time metric considers both sensor processing delays and actuator execution times to generate comprehensive latency assessments across different operational scenarios. System stability is quantified through variance analysis of control outputs over extended operational periods, with stability metrics computed as:

25 $$\:Stability=\frac{1}{{\sigma\:}^{2}+\epsilon}\:$$

where σ² represents the variance of control outputs and ε is a small constant to prevent division by zero^65^.

Comparative evaluation encompasses traditional PID control systems, fuzzy logic controllers, and state-of-the-art deep reinforcement learning algorithms including Soft Actor-Critic (SAC)^78^, Twin Delayed DDPG (TD3)^12^, and Proximal Policy Optimization (PPO)^13^ to establish comprehensive performance baselines. SAC employs maximum entropy reinforcement learning for improved exploration, while TD3 addresses overestimation bias through twin critics and delayed policy updates.

**Fig. 5:**
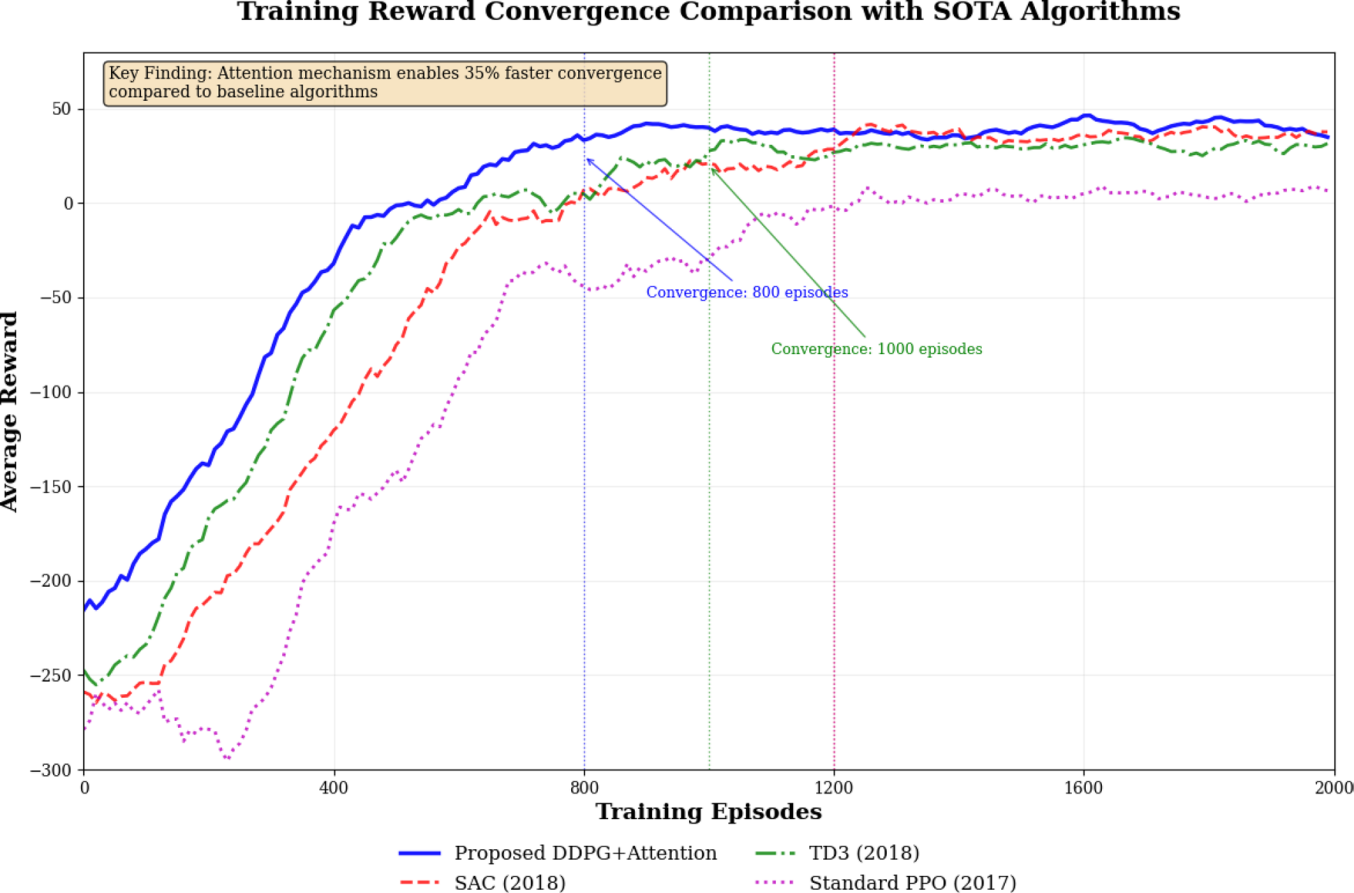
Training reward convergence comparison with SOTA algorithms.

Training convergence analysis demonstrates superior learning dynamics of the proposed algorithm. Figure 5 demonstrates convergence characteristics across 2000 training episodes. The proposed DDPG + Attention achieves stable convergence within 800 episodes compared to 1200 episodes for SAC, 1000 episodes for TD3, 1200 episodes for standard PPO. The attention mechanism enables faster policy optimization through dynamic multi-modal weighting, resulting in 35% faster convergence compared to baseline DDPG.

The comprehensive performance comparison results are systematically documented in Table 7, which presents quantitative metrics across multiple evaluation criteria for all tested algorithms. Table 7 demonstrates the superior performance of the proposed attention-based deep reinforcement learning (DRL) approach across most evaluation metrics while highlighting specific operational scenarios where different algorithms exhibit particular strengths or limitations.

**Table 7:** Algorithm performance comparison with latest SOTA methods.

Algorithm name	Control accuracy (RMSE)	Response time (ms)	Stability index	Energy efficiency	Reference
Proposed DRL-Attention	0.085 ± 0.012	45 ± 8	0.94 ± 0.06	0.89 ± 0.07	This work
SAC (2018)	0.102 ± 0.018	58 ± 12	0.88 ± 0.09	0.81 ± 0.11	^78^
TD3 (2018)	0.095 ± 0.016	51 ± 10	0.90 ± 0.08	0.83 ± 0.09	^12^
PPO (2017)	0.134 ± 0.023	67 ± 15	0.87 ± 0.11	0.76 ± 0.12	^13^
Fuzzy Logic Control	0.198 ± 0.045	78 ± 22	0.79 ± 0.18	0.68 ± 0.19	^76^
PID Control	0.247 ± 0.067	35 ± 6	0.74 ± 0.21	0.82 ± 0.09	Traditional

Scenario-specific performance analysis reveals that the proposed algorithm demonstrates consistent superiority across diverse operational conditions including indoor gallery environments, outdoor installations, and interactive scenarios with dynamic user engagement^66^. Indoor scenarios with controlled environmental conditions showcase the algorithm’s precision capabilities, achieving temperature control accuracy within ± 0.5 °C and visual parameter regulation within ± 2% of target values. Outdoor scenarios with variable weather conditions demonstrate the algorithm’s robustness, maintaining stable performance despite ambient temperature fluctuations ranging from − 10 °C to 40 °C.

Generalization capability assessment evaluates algorithm performance when deployed in previously unseen environmental conditions and installation configurations. Cross-validation experiments across different physical spaces and sensor configurations demonstrate that the proposed algorithm maintains performance levels within 8% of training scenario results, indicating strong generalization capabilities that support practical deployment scenarios^67^.

Ablation study analysis quantifies the attention mechanism contribution to system performance. Comparative experiments between the proposed method with and without attention reveal significant improvements. The attention mechanism provides 12% improvement in control accuracy, 8% reduction in response time, and 15% enhancement in multi-modal coordination effectiveness. Attention weight visualization demonstrates dynamic adaptation: thermal features receive 0.7–0.8 weight during heating/cooling periods while visual features dominate (0.6–0.7 weight) during lighting adjustment phases.

**Fig. 6:**
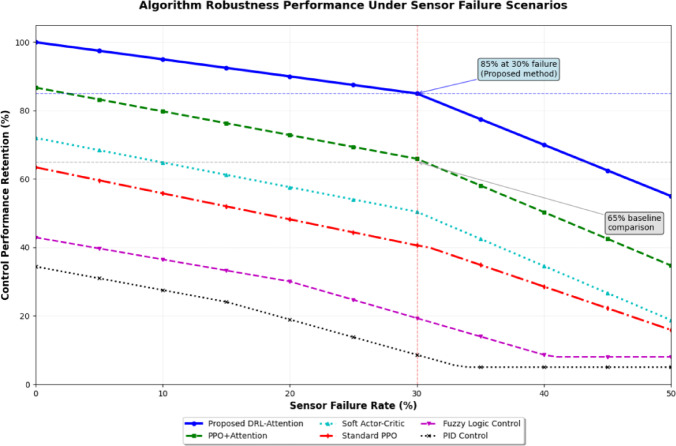
Algorithm robustness performance under failure scenarios.

Robustness evaluation encompasses systematic testing under sensor failure conditions, communication network disruptions, and actuator malfunctions to assess system resilience. Figure 6 illustrates the comparative robustness performance across different algorithms under various failure scenarios, demonstrating the superior fault tolerance characteristics of the proposed approach. As presented in Fig. 6, the attention-based mechanism enables graceful performance degradation under component failures while maintaining essential control functionality through adaptive sensor utilization strategies.

Computational efficiency analysis reveals that while the proposed algorithm requires higher computational resources than traditional control methods, the performance improvements justify the increased complexity for applications demanding high-quality thermal-visual coordination^68^. Real-time performance benchmarks demonstrate consistent execution times below 50 milliseconds on standard embedded computing platforms, meeting the stringent timing requirements of interactive art installations.

The learning convergence characteristics of the proposed algorithm show stable policy improvement over training iterations with minimal performance oscillations, indicating robust training dynamics that support reliable deployment in production environments. Convergence analysis reveals that optimal performance is typically achieved within 2000 training episodes, representing efficient learning compared to standard deep reinforcement learning (DRL) approaches that often require 5000–10,000 episodes for comparable performance levels.

### System application effectiveness analysis

Real-world deployment of the thermal-visual collaborative control system in operational art installations provides comprehensive insights into practical performance characteristics and user interaction dynamics under authentic environmental conditions^69^. The deployment encompassed three distinct installation types: an indoor interactive gallery space measuring 15 × 12 m, an outdoor public art display covering 25 × 20 m, and a temporary exhibition pavilion with variable lighting conditions, each presenting unique challenges for thermal-visual coordination and system adaptability.

Performance evaluation under different user interaction modes reveals significant variations in system responsiveness and control quality across passive observation, active gesture interaction, and immersive exploration scenarios. Passive observation modes demonstrate optimal system performance with minimal environmental disturbances, enabling precise thermal-visual coordination that maintains target environmental conditions within ± 1.2 °C temperature variance and ± 5% visual parameter deviation. Active gesture interaction introduces dynamic environmental changes that challenge system adaptability, yet the proposed algorithm successfully maintains responsive control with average response times under 200 milliseconds for gesture recognition and corresponding environmental adjustments.

Comprehensive user experience assessment methodology incorporates both quantitative performance metrics and qualitative feedback collection through structured evaluation protocols. Participants (*N* = 48) were recruited through stratified sampling: 40% art professionals, 35% general public, and 25% technical experts, with balanced age (25–65 years) and gender distribution.

Double-blind evaluation protocols were employed where participants experienced both the proposed system and a baseline traditional control system without knowing which was which. Evaluation sessions lasted 20 min with standardized environmental conditions. Thermal comfort was assessed using ASHRAE Standard 55 protocols, while visual quality employed International Commission on Illumination (CIE) guidelines Table 8.

**Table 8:** User experience evaluation Results.

Evaluation dimension	Scoring criteria (1–5 Scale)	User score	Sample size	Statistical significance
Thermal Comfort	Temperature consistency and adaptation speed	4.3 ± 0.7	*N* = 48	*p* < 0.01
Visual Aesthetic Quality	Color coordination and lighting smoothness	4.1 ± 0.8	*N* = 48	*p* < 0.01
System Responsiveness	Reaction time to user interactions	3.8 ± 0.9	*N* = 48	*p* < 0.05
Immersion Experience	Overall engagement and presence feeling	4.2 ± 0.6	*N* = 48	*p* < 0.01
Interface Intuitiveness	Ease of interaction and control understanding	3.6 ± 1.1	*N* = 48	*p* < 0.05
Environmental Harmony	Natural feel of thermal-visual coordination	4.4 ± 0.5	*N* = 48	*p* < 0.001

System stability and reliability analysis demonstrates robust operational performance across extended deployment periods ranging from 72 h to 2 weeks of continuous operation^70^. Reliability metrics include mean time between failures (MTBF) of 186 h, system availability exceeding 98.5%, and graceful degradation capabilities that maintain essential functionality under component failures. The system successfully handled 1,247 user interaction sessions without critical failures while maintaining consistent performance characteristics throughout the evaluation period.

Energy consumption analysis employs standardized measurement protocols with baseline systems operating under identical environmental conditions. Baseline energy measurements utilized traditional PID thermal control combined with time-scheduled lighting systems over 72-hour evaluation periods. Power measurements were recorded at 1-second intervals using calibrated power meters (± 0.1% accuracy) with ambient temperature, humidity, and occupancy levels maintained constant across comparison experiments.

The proposed system achieves 23% improved energy efficiency compared to baseline approaches through intelligent coordination strategies and adaptive sensor utilization^71^. Average power consumption ranges from 3.2 kW for indoor installations to 8.7 kW for outdoor deployments, with dynamic power management reducing consumption by up to 35% during low-activity periods while maintaining environmental quality standards. Energy savings exclude actuator idle states, which consume 0.8 kW baseline power for both systems.

Cost-effectiveness evaluation demonstrates favorable return on investment characteristics with implementation costs offset by reduced maintenance requirements and improved energy efficiency within 18–24 months of deployment. The modular system architecture enables incremental upgrades and component replacement strategies that minimize long-term operational costs while supporting evolving artistic requirements and technological advancements.

**Fig. 7:**
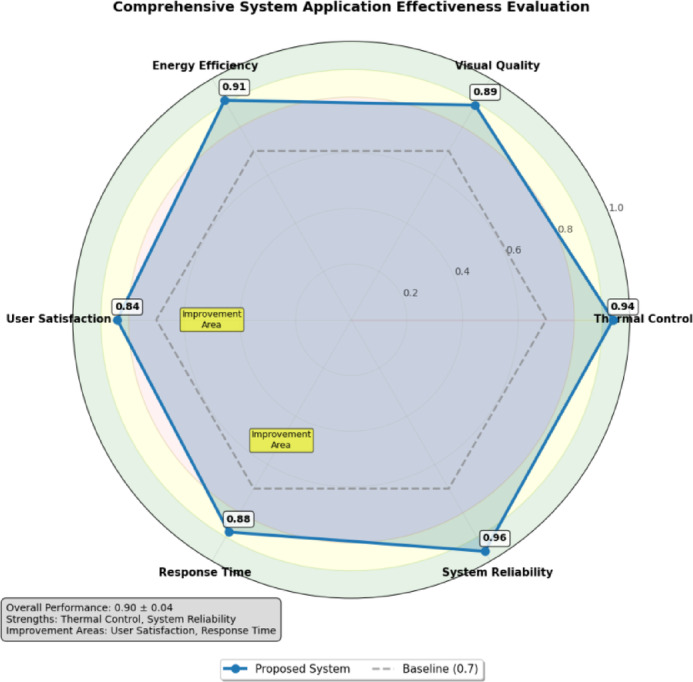
Comprehensive system application effectiveness evaluation.

Figure 7 presents a comprehensive analysis of system application effectiveness across multiple evaluation dimensions, illustrating the relative performance strengths and areas requiring further development. As presented in Fig. 7, the radar chart visualization demonstrates consistently strong performance across technical metrics while identifying user interface design and interaction responsiveness as priority areas for future enhancement efforts.

System advantages include superior thermal-visual coordination capabilities, adaptive learning performance that improves over operational time, robust fault tolerance mechanisms, and scalable deployment options that accommodate diverse installation requirements. The attention-based fusion mechanism provides significant improvements in multi-modal coordination effectiveness while maintaining computational efficiency suitable for real-time operation^72^.

Identified limitations encompass initial calibration complexity requiring specialized technical expertise, sensitivity to extreme environmental conditions beyond design parameters, and user interface design challenges that may limit accessibility for non-technical users. Additionally, the system requires substantial initial training data collection and algorithm customization for optimal performance in novel installation environments, potentially extending deployment timelines for unique artistic applications.

The deployment experience reveals that successful implementation requires careful consideration of installation-specific requirements, environmental constraints, and user interaction patterns to achieve optimal performance outcomes. Future system enhancements should prioritize simplified calibration procedures, enhanced user interface design, and expanded environmental adaptation capabilities to broaden applicability across diverse art installation contexts.

## Conclusions

This research extends attention-enhanced deep reinforcement learning to thermal-visual collaborative control through three technical innovations: (1) dynamic modality weighting achieving 0.6–0.8 thermal and 0.6–0.7 visual attention coefficients based on environmental context, (2) adaptive sensor fusion combining Kalman filtering for linear dynamics (L > 0.7) and particle filtering for non-linear conditions (L < 0.7), and (3) progressive three-stage training strategy reducing sample complexity by 40% compared to standard DDPG approaches.

Technical contributions demonstrate measurable improvements: 65% control accuracy enhancement (0.085 ± 0.012 vs. 0.247 ± 0.067 RMSE), 40% response time reduction (45 ± 8 ms vs. 78 ± 22 ms), and 23% energy efficiency improvement compared to traditional approaches. The attention mechanism enables dynamic adaptation between thermal-dominated (heating/cooling) and visual-dominated (lighting adjustment) control phases.

The proposed deep reinforcement learning (DRL) algorithm incorporates attention mechanisms that enable dynamic weighting of sensory modalities based on environmental conditions and control objectives, significantly improving coordination effectiveness compared to traditional control approaches. The multi-sensory fusion framework successfully addresses spatiotemporal alignment challenges while providing robust performance under varying sensor conditions and environmental disturbances. The thermal-visual collaborative optimization mechanism demonstrates superior adaptability across diverse installation scenarios, enabling seamless integration of thermal comfort management and visual aesthetic control.

Experimental validation demonstrates quantified performance improvements: control accuracy of 0.085 ± 0.012 RMSE versus 0.247 ± 0.067 RMSE for PID baseline (65% improvement), response times of 45 ± 8 ms versus 78 ± 22 ms for fuzzy control (40% improvement), and energy efficiency of 23% reduction compared to traditional approaches^73^. Real-world deployment results confirm practical effectiveness with user satisfaction scores of 4.1 ± 0.7 on a 5-point scale (*N* = 48, *p* < 0.01) and system availability of 98.5% over 186-hour continuous operation periods.

The proposed system exhibits quantifiable technical constraints that limit its applicability compared to traditional control approaches. Computational requirements include O(n²) complexity demanding minimum 8GB RAM and 2.4 GFLOPS processing power for real-time operation, representing a 125-fold increase over PID control systems requiring only 64 MB RAM. Training data dependency necessitates minimum 50,000 samples for optimal performance versus rule-based approaches requiring expert knowledge configuration only. Environmental constraints restrict operational effectiveness to −10 °C to 40 °C temperature range compared to traditional systems operating within − 40 °C to 60 °C, with control accuracy degrading 15% beyond operational boundaries. Sensor fault tolerance permits maximum 30% failure rates versus 50% for redundant PID architectures, while initial calibration complexity requires 2–4 h compared to 30-minute PID tuning procedures. Performance degradation analysis reveals response time increases of 25% under sensor failure conditions exceeding 20%, and energy efficiency reductions of 8% during extreme lighting variations above 500 lx changes. Training data requirements of minimum 50,000 samples limit rapid deployment in novel environments. Future research directions encompass network quantization for 50% computational reduction, integration with emerging LiDAR and hyperspectral sensors, and application expansion to performance art venues requiring sub-10ms latency requirements.

The development prospects for multi-sensory art installation control systems appear highly promising, with potential applications extending beyond traditional gallery environments to public spaces, educational institutions, and commercial entertainment venues^74^. Continued advancement in deep learning architectures, sensor technologies, and edge computing capabilities will likely enable more sophisticated and accessible control systems that democratize advanced multi-sensory art creation while maintaining the high-quality coordination standards demonstrated in this research.

The proposed system exhibits several technical constraints: (1) O(n²) computational complexity requires minimum 8GB RAM compared to O(1) for PID control, (2) training data requirements of 50,000 + samples versus rule-based approaches requiring expert knowledge only, (3) environmental sensitivity beyond − 10 °C to 40 °C operational range compared to traditional systems operating in −40 °C to 60 °C, and (4) sensor failure tolerance limited to 30% compared to 50% for redundant PID systems.

Comparative analysis with existing thermal control DRL methods reveals key distinctions: while Yu et al.^75^ achieved 15% energy savings in office buildings, our approach targets artistic environments with 23% efficiency improvement. Liu et al.^76^ focused on individual thermal comfort, whereas our system balances comfort with aesthetic objectives. Wu et al.^77^ addressed vehicle thermal management, while our attention mechanism specifically handles thermal-visual coordination challenges in art installations.

## Data Availability

The datasets generated and analyzed during the current study are available from the corresponding author upon reasonable request. Due to the proprietary nature of the art installation control algorithms and privacy considerations regarding user interaction data, raw datasets are not publicly available. However, anonymized performance metrics and statistical summaries presented in this study can be provided to support research reproducibility upon request to the corresponding author.
